# Development of a national survey on foot involvement among people with psoriatic arthritis in Australia using a best practice approach: a survey development protocol

**DOI:** 10.1186/s13047-020-00424-w

**Published:** 2020-08-26

**Authors:** Kate Carter, Steven Walmsley, Keith Rome, Deborah E. Turner

**Affiliations:** 1 Podiatry department, School of Health Science, Western Sydney University, Campbelltown Campus, Sydney, Australia; 2 Health and Rehabilitation Research Institute, Faculty of Health and Environmental Science, AUT University, 90 Akoranga Drive, Northcote, Auckland, 0627 New Zealand; 3 Podiatry department, School of Clinical Sciences, Kelvin Grove Campus, Queensland University of Technology, Brisbane, Queensland Australia

**Keywords:** Psoriatic arthritis, Foot, Survey research, Survey protocol, Patient-reported outcome

## Abstract

**Background:**

Limited research to date has defined the nature and extent of foot involvement in a psoriatic arthritis-specific population in Australia and the scale of the problem remains unclear. Survey research provides the ideal opportunity to sample a large population over a wide geographical area. Although quality criteria for survey research have been developed, research shows that adherence is low and that survey studies are poorly reported in peer-reviewed survey articles, which limits the ability to inform future survey design. The objective of this paper was to develop a national survey about foot involvement in people with psoriatic arthritis using a best practice approach. This is a methods paper for the development of survey research.

**Methods:**

A systematic, multi-stage process of survey development was undertaken, which comprised 3 phases: 1) the generation of the conceptual framework and survey content; 2) the development of the survey and pre-testing and 3) development of the survey dissemination strategy. A survey best practice approach was adopted using iterative pre-testing techniques, which included; cognitive debriefing, cultural sensitivity review, survey design expert validation, subject expert validation and pilot testing. Targeted postal and online survey dissemination strategies were developed a priori to optimise the response rates anticipated.

**Results:**

A 59-item survey with 8 sections was developed. Findings demonstrated a high survey response (*n* = 649), high data completeness (83% of respondents reached the end of the survey) and low rates of missing data (below 5% for 95% of respondents). Extensive survey pre-testing among the target population, health professionals and experts improved the overall quality, content validity, functioning and representativeness of the survey instrument, which optimised potential response rates. Clear audit trails that mapped the analytical process at each stage substantiated the rigour of the survey development methods. Robust strategies for sampling, survey dissemination and community engagement were deemed to have made a powerful contribution to response rates and the scale of information collected.

**Conclusions:**

Robust patient-centred methods in survey design were used to create a novel, high-quality survey to comprehensively evaluate psoriatic arthritis-related foot involvement. Transparent and precise description of the survey design and dissemination methods provides useful information to other researchers embarking on survey design in healthcare.

## Background

Psoriatic arthritis (PsA) is well recognised as a distinct clinical entity with a high disease burden [1–4]. Typically affecting people between 30 and 50 years old [5], PsA is associated with high economic and societal costs with over 25% of those at working age unemployed [6, 7]. For a large proportion of people with PsA, localised and persistent disease in the foot is their single most prevalent health complaint [8], which can have a profound impact on functioning and daily life [9]. Current knowledge of foot involvement in PsA is based on a few European studies, with limited incorporation of the patient perspective [10–13]. Despite recognition that hallmark disease features are predominant in the foot and ankle (such as enthesitis and dactylitis) [10, 14–16], foot involvement in PsA remains under-researched and poorly understood with a lack of large-scale data to provide the basis for targeted disease-specific assessments and interventions.

Survey research provides the opportunity to sample a large population over a wide geographical area and to measure a broad range of constructs with sufficient granularity [17, 18]. However, patient surveys have often been criticised for the lack of conceptual and methodological rigor [19]. Poorly designed surveys and inadequate reporting can lead to inappropriate application of research findings in decision-making, healthcare, health policy and future research [19–21]. To overcome this, quality checklists and reporting guidelines have been developed in order to promote complete and transparent reporting among researchers and to indirectly improve the comprehensiveness and credibility of survey studies [22–26]. The checklists include the SUrvey Reporting GuidelinE (SURGE) for paper-based surveys [25] and the CHEcklist for Reporting Results of Internet E-Surveys (CHERRIES) for web-based surveys [26]. Despite the development of guidance for reporting of surveys nearly a decade ago, previous reviews of published survey research have found that key quality criteria relating to design, conduct and results were under-reported in the majority of studies [19, 20]. In addition, there is limited empirical evidence for optimal survey design [27, 28]. It is well established that the methods used in conducting health surveys can significantly affect the reliability, validity and generalisability of study findings [28, 29] and that concordance with guidelines improves the quality of reporting research [30–32]. However, with major discrepancies in survey reporting identified [20] there are few high-quality worked examples of survey design and conduct to help researchers implement robust reporting practices. Therefore, the objective of this paper was to develop a national survey about foot involvement in people with PsA living in Australia using a best practice approach. In the absence of established empirical evidence on survey design, the current study methods were developed in accordance with best practice standards for the development of self-administered surveys [33, 34] and the subsequent description of survey conduct adhered to good reporting practices [20, 25, 26]. This is a methods reporting paper that provides a robustly designed method that can be replicated for future survey research. Whilst the current survey was created for research purposes and has not been used in clinical practice, the information it provides could yield important insights for a clinician that may not normally be considered; informing the holistic management of a person with PsA and helping to build a better understanding of the personal impact to better target care.

## Methods

### Study design

**Fig. 1:**
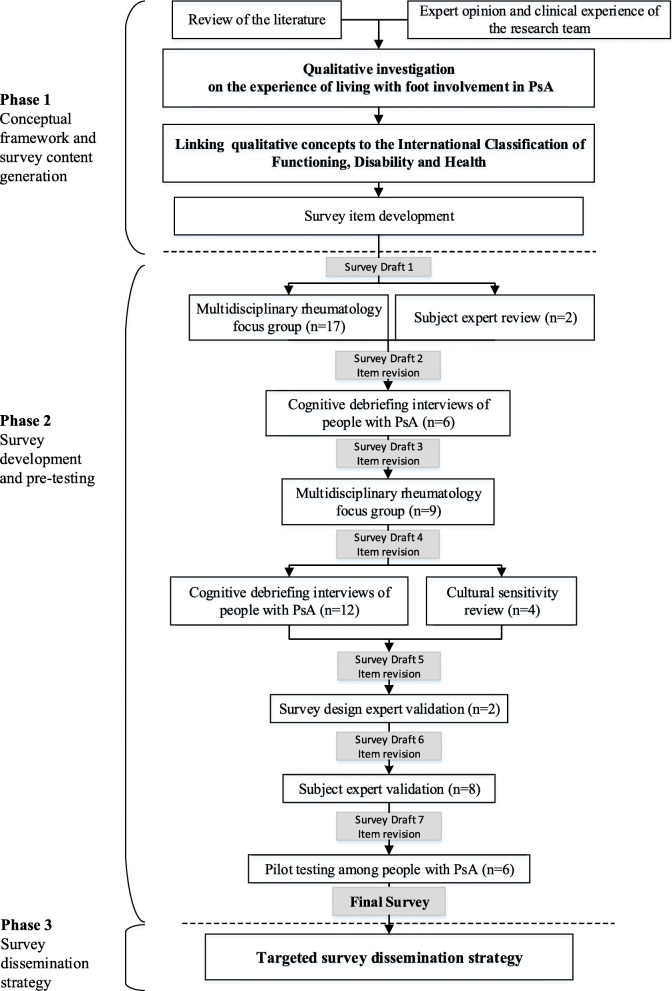
Survey development process. *PsA* Psoriatic arthritis

A cross-sectional observational study design was used to develop a self-administered paper-based and web-based survey (Fig. 1). Qualitative research methods were used to define and conceptualise important and relevant constructs for survey item generation (Phase 1). Pre-testing and piloting of the draft survey were intentionally timed to allow for analysis and revision of survey items, ultimately leading to 7 iterative revisions over an 18-month period (Phase 2). Major sites for dissemination were identified through a sampling strategy in order to determine the response rate relative to the target population (Phase 3) and the survey was disseminated and open for responses over a 6-month period.

### Participant recruitment

People with PsA and rheumatology health professionals involved in phases 1 and 2 were recruited using a convenience sampling technique with attention to ensuring diversity across health sectors and regions. A total of six sites across Australia and New Zealand were included, comprising three rheumatology public hospital outpatient departments, two university-based podiatry departments and a multidisciplinary rheumatology private practice with ethical approval granted for each participating site (refer to ethical approval statement). Written informed consent was provided by all participants prior to data collection.

Demographic and clinical information were collected from people with PsA for the purpose of describing the sample and have been previously reported for Phase 1 [9, 35] and are represented in Table 1 for Phase 2. Key demographic and practice details were collected for the health professionals (Table 2). All qualitative data collection in phase 1 and 2, (focus groups, interviews and reviews) were conducted by the principal investigator (KC) and supported by a second investigator (SW). Both investigators had experience of qualitative research methods and 15 years of clinical podiatry experience. Results were refined by discussion between the investigators (KC, SW and DET).

**Table 1:** Demographic and clinical characteristics of participants with psoriatic arthritis

Variables	Round 1	Round 2		Round 3	
	Cognitive interviews (*n* = 6) Paper-based	Cognitive interviews (*n* = 6) Paper-based	Cognitive interviews (*n* = 6) Web-based	Pilot testing (*n* = 3) Paper-based	Pilot testing (*n* = 3) Web-based
Ages, years	45 (15)	48 (12)	56 (14)	48 (9)	58 (12)
Women, n (%)	5 (83)	2 (33)	2 (33)	2 (67)	2 (67)
Ethnicity, n (%)					
Australian European	6 (100)	5 (83)	3 (5)	1 (34)	1 (34)
New Zealand European			1 (17)		
Indian		1 (17)	1 (17)		
South American			1 (16)	1 (33)	
British				1 (33)	1 (33)
Bosnian					1 (33)
Body Mass Index, Kg/m^2^	34 (12)	29 (6)	30 (8)	35 (11)	26 (2)
Employment status, n (%)					
Employed full-time	3 (50)	3 (50)	3 (50)	1 (34)	1 (34)
Employed part-time	1 (17)				1 (33)
Self-employed	1 (17)		1 (17)		
Unemployed (health reason)		1 (17)	1 (17)	1 (33)	
Unemployed (home-maker)		1 (17)			
Retired	1 (16)	1 (16)	1 (16)	1 (33)	1 (33)
Education level, n (%)					
No school certificate	1 (17)	1 (17)		1 (34)	1 (33)
School certificate	1 (17)			1 (33)	
Higher school certificate		2 (33)	1 (17)		
Trade/apprenticeship		1 (17)	3 (50)		
Diploma	2 (33)				
University degree or higher	2 (33)	2 (33)	2 (33)	1 (33)	2 (67)
Disease duration, years	15 (12)	15 (9)	5 (4)	14 (10)	3 (2)
Patient global assessment Joint & skin (VAS 0–100), mm	74 (15)	37 (37)	40 (24)	40 (38)	50 (26)
Skin (VAS 0–100), mm	36 (37)	28 (29)	25 (19)	7 (6)	37 (40)
Joint (VAS 0–100), mm	66 (25)	37 (35)	47 (16)	40 (36)	50 (26)
Global pain (VAS 0–100), mm	69 (21)	37 (36)	53 (21)	40 (26)	47 (38)
Foot pain (VAS 0–100), mm	73 (22)	37 (37)	38 (43)	33 (6)	60 (26)
Length of interview, minutes	63 (21)	60 (23)	6 (3)	*^2^	*^2^
Time to complete survey	*^1^	*^1^	29 (6)	26 (6)	22 (4)

Data presented as mean (SD) unless specified. *VAS* Visual analogue scale

*^1^ Participants were interviewed about the survey to obtain their views but did not complete it

*^2^ Following survey completion, participants were asked if they had experienced any difficulties. There was no audio-recorded interview

**Table 2:** Demographic characteristics of the health professionals with experience of managing people with psoriatic arthritis and subject experts

Variables	Round 1		Round 2	Round 3	Round 4	Round 5
	MDT rheumatology review (*n* = 17)	Subject expert review (=2)	Health professional focus group review (*n* = 9)	Cultural sensitivity review (*n* = 4)	Survey expert review (*n* = 2)	Subject expert review (*n* = 8)
Women, n (%)	11 (65)	2 (100)	4 (44)	3 (75)	1 (50)	3 (38)
Geographic location, n (%)						
NSW, Australia	10 (59)	2 (100)	9 (100)		1 (50)	3 (38)
QLD, Australia	3 (18)					
SA, Australia	3 (18)					
NT, Australia	1 (5)					
Auckland, NZ				4 (100)		1 (12)
United Kingdom					1 (50)	4 (50)
Occupation, n (%)						
Rheumatologist	4 (24)	1 (50)	1 (11)			3 (38)
Podiatrist		1 (50)	8 (89)	3 (75)		5 (62)
Physiotherapist	4 (24)					
Exercise physiologist	1 (6)					
Nurse	6 (35)					
Clinical researcher	1 (6)					
Pharmacist	1 (5)					
Maori research advisor				1 (25)		
Survey specialty					2 (100)	
Clinical experience, years	13 (13)	13.5 (9)	12 (10)	11 (10)	8 (1) *^2^	15 (13)
Health sector, n (%)				*^1^	*^3^	
Public sector	9 (53)		5 (56)			6 (75)
Private sector	5 (29)					
Mixed	3 (18)	2 (100)	4 (44)			2 (25)
Length of interview, minutes	53	45	57	66	*^4^	97

Data presented as mean (SD) unless specified. *PsA* Psoriatic arthritis. *ACT* Australian Capital Territory, *NSW* New South Wales, *NT* Northern Territory, *SA* South Australia, *QLD* Queensland, *NZ* New Zealand.

*^1^ Health professionals of the cultural sensitivity review were academic staff at Auckland University of Technology and not currently practicing in the health sector

*^2^ Not relating to clinical experience but to experience of survey development, evaluation and implementation

*^3^ The survey experts were not practicing in the health sector

*^4^ Written feedback was provided. There was no audio-recorded interview

### Survey development protocol

#### PHASE 1: generation of the conceptual framework and survey content

**Fig. 2:**
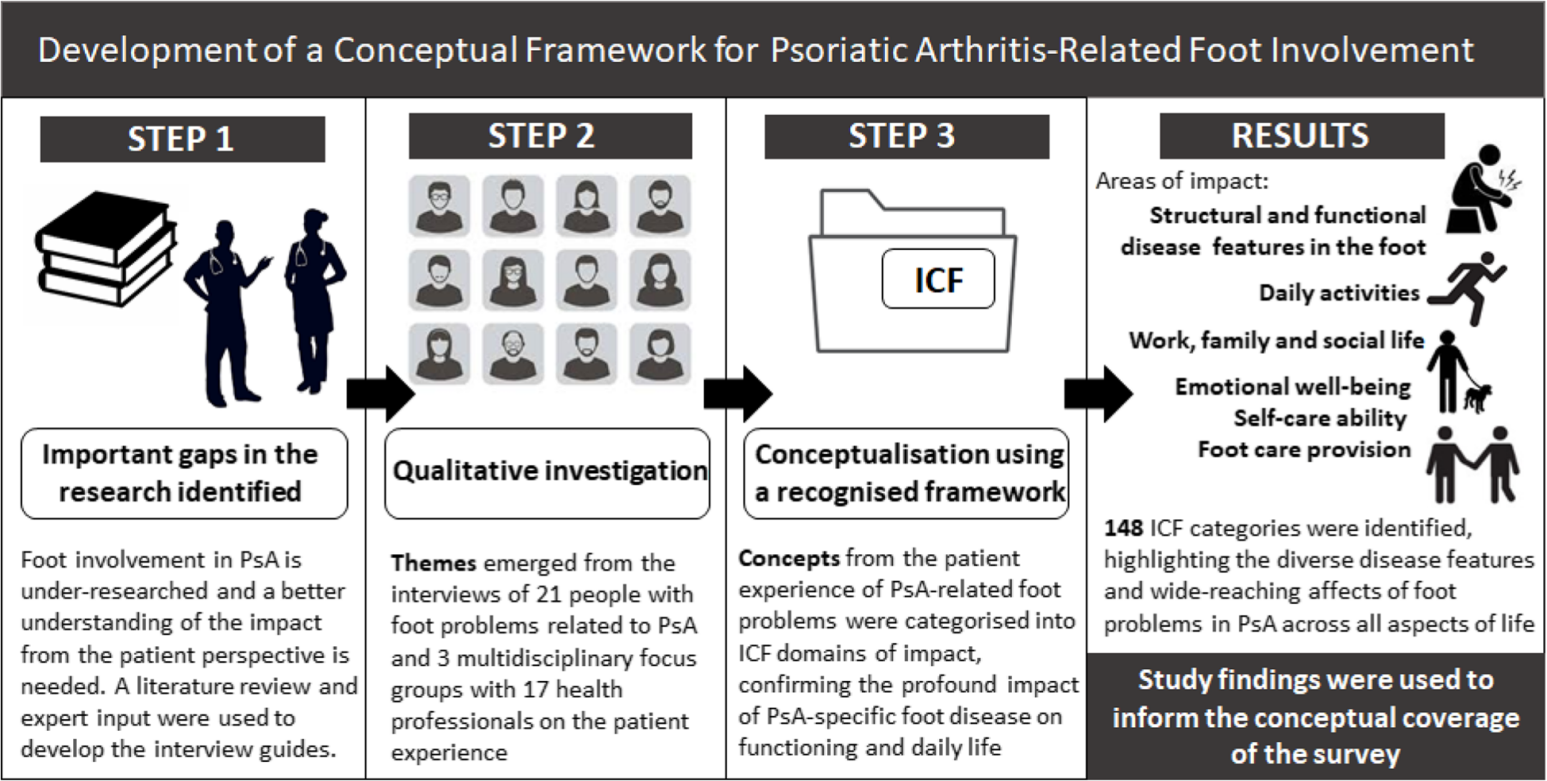
Development of a Conceptual Framework for Psoriatic Arthritis-Related Foot Involvement. (Adapted from Alam, et al., 2020 [37]). *PsA* Psoriatic arthritis, *ICF* International Classification of Functioning, Disability and Health

Detailed descriptions of the preliminary work involved in phase 1 have been previously reported [9, 35, 36]. In brief, a qualitative study of people with PsA and rheumatology health professionals (rheumatologists, podiatrists and physiotherapists) revealed key themes on the impact of PsA-related foot problems on daily life [9, 35]. Concepts important and relevant to the patient experience of foot involvement in PsA, obtained from the qualitative study, were subsequently linked to the International Classification of Functioning, Disability and Health (ICF) model and a list of over 100 distinct ICF categories were identified, which comprehensively described the impact of localised disease in the foot on daily functioning [36]. The qualitative research findings and ICF linking exercise provided the basis of which to inform the conceptual framework for the survey and subsequent item generation. Conceptual frameworks are developed to provide the theoretical underpinning for identifying what should be included when measurement tools are developed [37]. The conceptual framework served as the foundation for the subsequent stages of survey development (Fig. 2).

#### PHASE 2: survey development and pre-testing

Paper and online formats of survey administration were developed with the aim to increase response rates and reduce potential selection bias. Throughout the pre-testing phase survey content remained the same irrespective of the method of completion, with adaptions to survey item content reflected in both versions of the paper and web-based survey. The online version of the survey was developed using Qualtrics software [Qualtrics, Provo, UT, USA].

Pre-testing is the process of collecting validity evidence to support and ensure the content, cognitive and usability standards of the final survey [34, 38]. A combination of pre-testing methods was used to obtain critical appraisal from multiple perspectives in order to increase the likelihood of success for the final survey [27, 34, 39]. The survey design protocol comprised 6 pre-testing techniques:

1. focus groups with health professionals in rheumatology,
2. cognitive debriefing interviews of people with PsA,
3. cultural sensitivity review with New Zealand-based health professionals,
4. survey design expert validation,
5. content expert validation,
6. pilot testing among people with PsA.

Qualitative feedback from pre-testing sessions was used to make inclusion, exclusion and revision decisions for individual items, with data tabulated and examined for patterns in responses to each question [40]. A clear audit trail of survey revisions and refinements was recorded in order to ensure the comprehensive integration of responses from pre-testing sources and document the refinement of the survey (summarised in Supplementary Materials).

##### Focus groups with health professionals in rheumatology

Multidisciplinary rheumatology focus groups systematically evaluated the survey content in order to make improvements to the overall quality and representativeness of the items. Health professionals experienced in the management of people with PsA were recruited. A total of 2 focus groups (participant number range 9–17) were conducted, which is in accordance with recommended numbers needed to generate consensus on proposed scale items [41]. Health professionals in the first focus group were recruited from a national Australian rheumatology conference in 2018 with diversity of participants from different states and territories, professions and health sectors. Health professionals in the second focus group were recruited from an outpatient rheumatology and podiatry hospital department in Sydney, Australia (Table 2). An interview guide ensured discussion on priority areas including the acceptability of each item, anticipated responder burden, relevance of items, clarity of instructions and overall survey coverage. All focus groups were audio-recorded, transcribed verbatim and analysed using qualitative data matrices to categorise responses [42]. Survey items were revised and reduced based on data from each focus group.

##### Cognitive debriefing interviews

In-depth cognitive debriefing interviews of people with PsA were used to determine how respondents comprehend questions, process and recall information and decide what answers to give, which identified potential sources of error, such as with the interpretation of items and response anchors [43–45]. An interview script was designed using a standardised protocol that involved the think-aloud technique with concurrent verbal probes [46]. A total of 18 people with PsA-related foot problems participated in three rounds of cognitive interviews, with equal participant numbers in each round. Two rounds were undertaken using the paper-based survey and one round the web-based version. All cognitive interviews were audio-recorded, transcribed verbatim and analysed using qualitative data matrices to categorise and interpret responses [43]. The survey was modified and improved based on feedback from each round of cognitive debriefing, which related to the categories outlined in Table 3.

**Table 3:** Definitions of the categories used to assign responses from the cognitive debriefing interviews, focus groups and expert reviews in order to organise decisions for survey item revision

Categories	Description	Example(s)
Poor wording	Word changed or spelling error	Do you see any errors in wording? For example, ‘crocked’ changed to ‘crooked’, ‘ethic group’ changed to ‘ethnic group’, remove the word ‘hobble’
Comprehension and interpretation	Ability to understand the question, to accurately interpret its meaning and to follow the item instructions	What does ‘anxiety’ and ‘depression’ mean to you? Can you tell me, in your own words, what the instructions are asking you to do? Can you repeat this question in your own words?
Judgement	Ability to make considered decisions	How confident are you that you are able to mark accurately where you have or had experienced pain on the diagram? How confident are you that you can remember how many times you fell over the past 12 months?
Navigation	Navigate features of the survey and progression through the survey	Is it easy or hard to scroll to see the questions? Would you like the option to go back and review or edit your responses?
Timescales	Appropriate timescales used, acceptable recall periods	Is it easy or hard for you to remember when your symptoms first started? Do you feel that the timescale of this question is appropriate
Redundancy and repetition	Survey item is not required, no longer useful, or is too similar to another item	Do you think that any of the questions are repetitious? Is this question relevant to you?
Response options	Acceptable number and range of response options	Do you think that the answers you can choose from allow you to answer the questions in the way that you want?
Emotiveness	Triggers an unwanted emotional response	How does answering this question make you feel? For example, sad, frustrated, uncomfortable?
Responder burden	Number of survey items, time taken to complete the survey, survey length	Do you think that the respondents will have the motivation, knowledge and ability to answer the questions? Do you think the length of the survey is burdensome?
Unclear purpose	Survey items collecting data that do not appear to alignment with the research purpose	For example, collecting information about global disease is not related to the purpose of the survey about foot problems, explanation required.
Missing information	Information not already captured within the survey	Suggestion to add a question: To find out if patients access services in the public or private settings To identify the impact of proximal issues on the foot and mobility
Cultural sensitivity	Cultural factors that affect the functioning of the survey in a different country	Can you think of any problems or issues that patients in New Zealand might encounter? Do you feel that the survey has reasonable cultural sensitivity (taking into account the cultural and language differences between Australia and New Zealand) and can be adapted for people with psoriatic arthritis living in New Zealand? For example, the wording of different types of footwear will be different between countries
Face and content validity	Sufficient coverage of items, meaningful to patients	Does the survey consist of a broad range of items that are all relevant, in their coverage, to the nature, extent, location and impact of psoriatic arthritis-related foot involvement on patients’ daily lives? Does the survey appear, on the face of it, to measure the problems you have with your feet and the impact it has on your life?

The patient pre-testing was valuable for managing concerns raised by health professionals on terminology complexity and the belief that people with PsA would need simpler alternatives to some medical terms in order to aid understanding. Indeed, comprehension and interpretation concerns were continually raised during survey item revisions by health professionals. Whilst some items were simplified to account for varying levels of understanding, the majority of people with PsA were familiar with technical language such as plantar fasciitis, dactylitis and pitting nails and the cognitive debriefing interviews supported the respondents’ understanding of each item.

As part of the cognitive debriefing interviews for the web-based survey, usability pre-testing involved participants completing the survey on their own mobile devices (smartphone or tablet) or other electronic devices (home computer or laptop). The evaluation of the web-based survey included all the elements of the paper version, but participants were additionally asked to comment on the online layout and design of the survey items and navigation features. Web-based design modifications were introduced as a result in order to optimise the user experience and ensure response options would display correctly on different types of electronic devices [47, 48].

##### Cultural sensitivity review

The original research intention was to conduct the survey in both Australia and New Zealand. However, challenges related to ethical requirements, logistical management and time restrictions, resulted in the New Zealand arm of the study being postponed. However, for completeness, the cultural sensitivity review is presented. For both national and international multicentre studies, cultural sensitivity reviews are required to identify and correct for any cultural differences in the interpretation of survey items [28]. In Australia, pre-testing was conducted among people with PsA and health professionals from different health care settings and socioeconomic locations to ensure that a wider range of opinions were collected. In New Zealand, in order to assess the usability and cultural sensitivity of the survey in the local context a focus group was conducted, which included 3 health professionals with expertise in podiatry-rheumatology research and 1 Māori research advisor. The majority of changes made to the survey related to language (in order to reflect local terminology) and footwear differences between Australia and New Zealand.

##### Survey design expert validation

Experts (*n* = 2) in the development and evaluation of healthcare surveys and patient-centred outcome research reviewed the draft design of the paper and web-based survey. Feedback was provided in the form of detailed free-text annotations on the survey and written comments to specific questions on how to optimise the look, flow and design of the survey. Consequently, survey items were re-grouped, streamlined and survey sections were numbered to improve consistency in navigation and layout between the paper and online versions of the survey.

##### Subject expert validation

An 8-member specialist panel, including 4 international leading experts in PsA-related foot involvement and 4 members of the research team (DET, SW, KR, MO), reviewed the final survey items and response scales. The panel members were selected based on a priori criteria, which included having recent publications in PsA-specific foot involvement research, a track record of special interest in PsA and current specialised clinical practice in PsA. An innovative online collaborative platform was used to facilitate the instant recording of responses, while web-conferencing allowed real-time audio/visual communication between panel members across 3 countries including the UK, Australia and New Zealand. Panel members independently rated the relevance and importance of each item to its assigned construct; demographics, pain and musculoskeletal disease, skin and toenail disease, function and participation, footwear, treatment burden and emotional well-being. The review process was moderated (SW) in order to ensure that all panel members had rated and commented on each survey construct and progressed as a group. Following independent, real-time completion of the data form, results were presented to all members for open discussion in order to reach a consensus on the final survey items. To ensure face and content validity, the revised items were then re-reviewed by the experts to confirm that they were acceptable for inclusion. The expert panel recognised the need for all items given the heterogeneity of disease expression and potential impact.

##### Pilot testing

Pilot testing is a ‘dry run’ of the entire survey administration; the target population completes the survey in the planned delivery mode and final revisions to the survey process are made ready for full-scale administration [39]. Six prospective participants completed the final 59-item survey, consisting of 8 sections, in the planned mode of delivery; 3 paper-based surveys were self-administered in a clinical setting, and 3 web-based surveys were self-administered at home. Both versions of the survey took between 20 to 25 min to complete. The pilot testing confirmed that respondents could complete the survey effectively, efficiently and found the survey length acceptable. This finding was in contrast to the view of the majority of health professionals in the focus groups who reported that the length of the survey was likely to be prohibitive to potential participants. In addition, the data analysis plan was informed by the pilot test results by prompting decisions for data entry, coding and handling [39].

### PHASE 3: survey implementation

Reporting survey implementation and dissemination is crucial in order to determine the generalisability of study results [20]. Survey development reporting guidelines (SURGE and CHERRIES) were used to report the survey development, implementation and data management for the paper and web-based surveys respectively [25, 26].

With PsA prevalence in Australia unknown a priori and in the absence of national databases to estimate the potential target population and response rate, the sampling strategy was to identify target estimates from the major sites for dissemination in order to establish the potential reach of survey to people with PsA. The estimated total target population was 6000 people with PsA in Australia. Targeted sites and organisations for dissemination of the survey were pre-identified. The survey was promoted by; 13 patient support organisations, 5 social media support groups, 7 health professional associations, 6 specialist clinical services and 2 research centres. Both the web-based and paper-based versions of the survey were run concurrently to optimise data collection. In total, 650 paper surveys were posted to the targeted patient community groups and specialist clinical services in Australia, as well as 2000 flyers and 300 posters. Consent was implicit by the return/submission of the completed survey and participants were informed of this at the start of the paper and online survey.

A study website, Facebook page, video and animation were created to generate research interest through online health social networks and communities, and to provide ongoing engagement through the survey dissemination phase. This targeted approach to survey dissemination was followed by snowballing and crowdsourcing sampling techniques, where participants informed other potential participants of the research by liking posts and sharing links about the survey. Monthly progress and reminder emails were sent to all target sites and organisations during the dissemination phase (6-months).

## Results

The final 59-item self-administered survey was developed based on feedback from each of the stages involving people with PsA, health professionals and experts. Despite the substantial length of the survey, the majority of people with PsA interviewed during survey pre-testing suggested that it was acceptable, which contrasted with the view that most health professionals had that the survey length would be prohibitive to potential participants. Reasons for this included 1) having a desire to share their experience of foot problems that they felt had been neglected and/or poorly understood, and 2) resonance of patient-derived statements throughout the survey, which reportedly had a positive effect on encouraging survey completion. Key survey domains included demographic (10%) and socioeconomic data (10%), global disease information (18%), foot and ankle characteristics (18%), and the impact of foot problems on daily life including daily routine, footwear choice, family life, work and accessing health care (44%). The percentage coverage of items directly reflects the dominant concerns of people with PsA-related foot problems and health professionals in rheumatology.

**Fig. 3:**
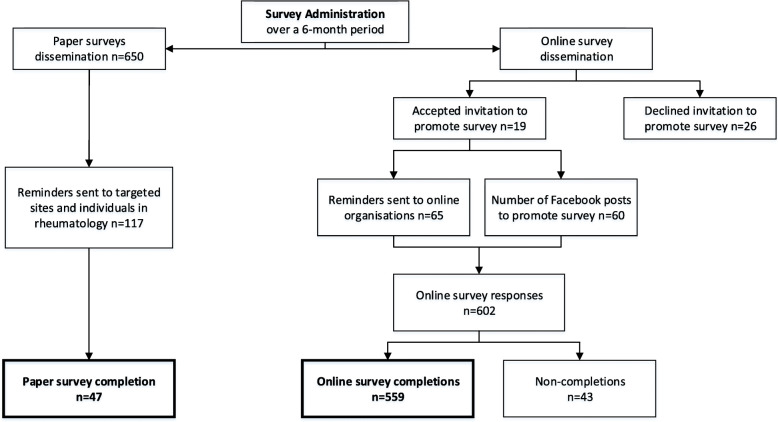
Flow diagram of survey dissemination

Quantity-related success outcomes included a total of 606 survey completions (10% response rate) that comprised 559 (92%) online survey completions and 47 (8%) completed paper surveys (Fig. 3). Of the 602 unique online views of the survey, 43 participants did not progress beyond the first information page or submit any data and were removed from subsequent analysis. The majority of respondents progressed through 100% of the survey to reach the end (83%). Valid and missing data for each survey item was evaluated in order to determine data completeness. The missing data for online and paper survey completions was below 5% for the majority of respondents (95%). Time taken to complete the online survey was a mean (SD) 21 (8) minutes. The majority of survey respondents (82%) indicated that they would like to be contacted again about future studies related to this research, which suggests high levels of engagement and an overall positive survey experience.

## Discussion

This paper provides an overview of the development of a survey on foot involvement in PsA based on best practice methods in survey design. Extensive pre-testing among key relevant stakeholder groups improved the overall quality, functioning and representativeness of the survey instrument. Although there is limited empirical evidence and few universal best practice recommendations for survey design and conduct [27–29], comprehensive and transparent descriptions of survey design methods can allow clearer review of the usefulness and validity of the survey research [20]. Despite the substantial length of the survey, high response and levels of data completeness suggest that the incorporation of insightful and meaningful concepts, generated by those with the disease, resonated with survey respondents and had a positive effect on survey completion. Engaging people with PsA, health professionals and experts in the survey development methods ensured that content and face validity were achieved and that all items together comprehensively reflected the construct being measured [49]. Extensive pre-testing confirmed that the survey possessed sufficiently high cognitive and usability standards for potential respondents to effectively engage with and successfully complete the survey. Undertaking several rounds of cognitive interviews among people with PsA in order to revise and refine the survey content and design was considered to be the most impactful of the pre-testing methods in achieving a good response, and other researchers with less time and resources should consider this an essential part of the method in survey development research.

Sample size requirements of this study were difficult to estimate in the absence of national patient databases and with unknown PsA prevalence in Australia. The total target population was estimated from the major sites for dissemination to be 6000 people with PsA (10% response rate). Similar New Zealand-based podiatry surveys received 197 web-survey completions from people with inflammatory arthritis (49% response rate, *n* = 400 target sample) [50] and 131 postal survey completions (32% response rate, *n* = 400 target sample) were received from people with systemic lupus erythematous [51]. Although New Zealand has a smaller general population than Australia, based on these existing published sample sizes [50, 51], the current study survey response of PsA-specific participants exceeded previous target samples by a substantial degree. Of the 43 participants who did not progress beyond the first information page to enter the survey, a proportion may have been other researchers, health professionals and administrators promoting the study that previewed the online survey and inadvertently contributed to the number of non-completions.

Patient self-report is increasingly used to assess the impact of PsA, to gain insight into the patient experience and to formulate new questions for investigation [52–54]. In accordance with international working groups in PsA [55–57], this large national survey has incorporated the patient’s voice in the measurement of localised disease impact by engaging patients in research activity throughout the survey development protocol. Discordance between the views of people with PsA and health professionals were identified in phase 2 of survey development, which related to medical terminology use and the length of survey. These findings were consistent with similar research [58], and emphasises the importance of embedding the patient perspective in the development of measures in PsA.

Limitations of the survey development process (phase 1 and 2) include a potential sampling bias with all research participants chosen by convenience sampling, meaning those who volunteered to take part in the survey development may not be representative of all people with foot involvement in PsA or the health professionals who manage this patient group. However, participants were recruited from multiple sites in order to achieve a diverse cross-section of the sample and recruitment continued until qualitative data saturation was reached. A limitation to the study generalisability is that the multiple iterations of pre-testing and pilot testing required to develop a high-quality survey are resource and time intensive, which may be prohibitive for some research teams. Future work will involve adaption and implementation of the survey in the UK. The findings from the programme of survey research will also be used to inform the development of an evaluative PsA foot-specific patient-reported outcome measure for use in clinical practice and research.

## Conclusions

This paper describes a robust survey development protocol using best practice methods in survey design and conduct, with the intent that this could be utilised as a framework for survey development in other areas of clinical practice. Involvement of people with PsA, health professionals and experts throughout the survey development process was a central component that ensured the survey functioned properly and yielded successful survey outcomes. Focus on high-standards of reporting survey research permits wider application of the protocol beyond the intended target population of the survey. Whilst the survey has not been used in a clinical setting with the purpose to better direct assessment and targeted care, future work is planned to determine the feasibility of its use to support assessment in clinical practice.

## Supplementary information

**Additional file 1.** Survey item revisions and refinements audit trail.

## Data Availability

The datasets used and/or analysed during the current study are available from the corresponding author on reasonable request.

## Abbreviations

ICF: International Classification of Functioning, Disability and Health
PsA: Psoriatic arthritis
VAS: Visual analogue scale
