# Supplementary material for: Development of a national survey on foot involvement among people with psoriatic arthritis in Australia using a best practice approach: a survey development protocol
Source: J Foot Ankle Res. 2020 Aug 26;13:53. doi: 10.1186/s13047-020-00424-w (PMC7448479; doi:10.1186/s13047-020-00424-w)
Supplement: Supplementary file 1 — Additional file 1. Survey item revisions and refinements audit trail. [file 13047_2020_424_MOESM1_ESM.docx]

**Supplementary Materials**

Figure 1 of 7. Process and outcome of the revision and refinement of the paper-based survey draft 1 based on the multidisciplinary rheumatology focus group and subject expert review.

Figure 2 of 7. Process and outcome of the revision and refinement of the paper-based survey draft 2 based on cognitive interviews of people with psoriatic arthritis-related foot involvement.

Figure 3 of 7. Process and outcome of the revision and refinement of the paper-based survey draft 3 based on the focus group with health professionals.

Figure 4 of 7. Process and outcome of the revision and refinement of the paper-based survey draft 4 based on cognitive interviews of people with psoriatic arthritis-related foot involvement and the New Zealand cultural sensitivity review.

Figure 5 of 7. Process and outcome of the revision and refinement of the web-based survey draft 4 based on the cognitive interviews of people with psoriatic arthritis-related foot involvement.

Figure 6 of 7. Process and outcome of the revision and refinement of the survey draft 5 based on the survey design expert review.

Figure 7 of 7. Process and outcome of the revision and refinement of survey draft 6 based on the subject expert review resulting in the final survey instrument (draft 7). Pilot testing of the paper-based survey (n=3) and the web-based survey (n=3) among people with psoriatic arthritis followed the process outlined above, and no further changes were made to the survey thus resulting in the final survey draft.
